# One-dimensional lead iodide hybrid stabilized by inorganic hexarhenium cluster cations as a new broad-band emitter†

**DOI:** 10.1039/d1ra04170c

**Published:** 2021-07-13

**Authors:** Giang Thi Ly, Jun Choi, Youngmee Kim, Yuna Kim, Sujin Kim, So-Hyeon Yang, Sung-Jin Kim

**Affiliations:** Department of Chemistry and Nanoscience, Ewha Womans University Seoul 03760 Republic of Korea sjkim@ewha.ac.kr

## Abstract

A novel one-dimensional (1D) hybrid {[Re_6_S_8_(PzH)_6_][Pb_3_I_8_(DMF)_2_]}·6(DMF) with hexarhenium cluster cations has been synthesized and characterized by means of single-crystal X-ray diffraction. Two DMF oxygen atoms bridge three lead iodides to form a set of lead iodides, {[PbI_4/2_I(O_DMF_)_1/2_][PbI_4/2_(O_DMF_)_2/2_][PbI_4/2_I(O_DMF_)_1/2_]}^2−^, and these sets of lead iodide share edges to form a 1D lead iodide chain, [Pb_3_I_8_(DMF)_2_]^2−^ which has never been reported before and is different from the typical edge sharing of octahedral PbI_6_ units. 1D lead iodide chains are stacked along the *a* axis, and [Re_6_S_8_(PzH)_6_]^2+^ cations with H-bonded DMF molecules to pyrazole N–H reside between 1D lead iodide chains. This 1D lead iodide hybrid shows strong broad-band emission with a peak at 634 nm. The excellent photoluminescent properties of the new lead iodide hybrid exhibit great potential for optoelectronic applications in photonic devices with broad-band emission and stability. This study introduces a new class of lead iodide hybrid compounds having new inorganic cluster cations rather than the organic amine cations that have been used in numerous studies to date. This work opens a promising path to overcome the instability of perovskites including of organic amine cations.

## Abstract 2

A novel one-dimensional (1D) hybrid {[Re_6_S_8_(Pz)_6_][Pb_3_I_8_(DMF)_2_]}·6(DMF) with hexarhenium cluster cations has been synthesized, and it shows strong broad-band emission with peak at 634 nm.

## Graphical abstract

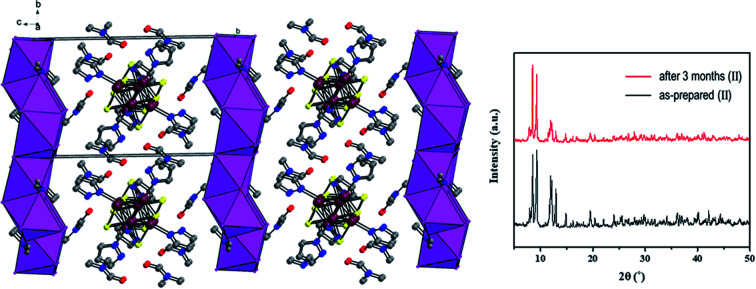

## Introduction

In recent years, research on organometal halide perovskites for optoelectronic and photovoltaic use has grown exponentially.^1–4^ The use of inorganic–organic hybrid perovskites is currently receiving a great deal of attention due to their superior light harvesting, tunable band gap, high color purity and low-cost solution processability.^5–8^ These perovskites can be classified depending upon the connectivities of their inorganic networks. The general formula for a three-dimensional (3D) perovskite is ABX_3_, where A is methylammonium (MA^+^), formamidinium (FA^+^), or Cs^+^; B is Pb^2+^, Sn^2+^, or Ge^2+^; and X is a halide (Cl^−^, Br^−^, or I^−^). 3D perovskites consist of corner-sharing metal halide octahedra, and A^+^ cations reside in the cavities of 3D inorganic frameworks. When larger organic cations than MA^+^ are employed, it is possible to form low-dimensional perovskite structures in which the connectivity of the inorganic network is reduced to two-dimensional (2D) sheets, one-dimensional (1D) chains, or zero-dimensional (0D) clusters.^4,9^ Lowering the dimensionality to 2D gives rise to increased structural diversity. The connectivity modes of the [BX_6_]^4−^ make the formula repeating unit of one-dimensional chains vary when the dimensionality is reduced from 3D or 2D to 1D. To date, face-sharing connectivity has been the most common connectivity in the 1D perovskite family of structures, followed by corner-sharing, and edge-sharing connectivity is the rarest structure in the 1D group. In general, the metal halide octahedra in 1D perovskites are connected in chain structures and these chains are wrapped by organic cations. The reduction of dimensionality in these perovskites greatly affects their optoelectronic and physical properties, opening a door to new realms of materials science.

In the near future, the ideal replacement technology for conventional incandescent and fluorescent lamps is light-emitting diodes (LEDs), because of their energy savings, high luminous efficiency, and long lifetime. Recently, organometal halide perovskites have emerged as a new class of materials for LED applications.^10–12^ In 2014, the first LED based on a perovskite material was reported by Friend and coworkers.^13^ This device showed a maximum external quantum efficiency (EQE) of 0.1% in the green region and 0.76% in the infrared region. In 2018, four different groups achieved EQEs greater than 20% in LEDs based on perovskite materials.^10–12,14^ Thus, perovskites have great potential for lighting applications. White light-emitting diodes are of special interest, and LEDs have numerous merits over conventional light sources.^15–17^ Diverse device architectures have been researched toward the goal of pure white emission. These architectures consist of (I) a blue LED chip, (II) a monochromatic blue LED, and (III) a single broad-band white phosphor LED.^18–20^ Poor color rendition, efficiency loss, and instability of emission color over time are serious drawbacks of approaches (I) and (II). Compared with these approaches, approach (III) has emerged as a rising star because it can overcome these difficulties. Low-dimensional perovskites are garnering more interest because of their structural tunability, leading to controllable band structure and tunable color. Compared with the research focus on optimization for 2D perovskites, less research attention has been paid to the 1D perovskites even though 1D perovskite chains also feature intrinsic broad-band light emissions. For example, Yuan and coworkers synthesized new 1D lead bromide perovskites that showed broad-band emission with a photoluminescence characteristic peak around 480 nm.^21^ 1D perovskites usually demonstrate stronger broad-band emission than 2D perovskites. However, most 1D perovskites to date have exhibited hypsochromically shifted white-light emission, with emission peaks ranging from 450 to 550 nm.^22^ Therefore, the synthesis of various bathochromically shifted 1D perovskite materials is desirable to prepare candidates for UV LEDs employing a single broadband white phosphor.

Until now, the ligands used to prepare low-dimensional perovskites have been large organic ammonium ligands such as C_4_H_9_NH_3_^+^, CH_3_(CH_2_)_8_NH_3_^+^, and 3-(aminomethyl)piperidinium (3AMP^+^).^23^ Herein we report a crystalline 1D lead halide containing a very new inorganic cation for this purpose, a hexarhenium cluster. Rhenium cluster compounds are known to exhibit broad emission windows from 550 to 1000 nm. These compounds are luminescent both in the solid state and in solution, and can be phosphorescent in the red and near-infrared regions, with long-lived microsecond-scale emission upon excitation by UV-Vis light.^24–33^ Although perovskite materials are desired for light-emitting device applications, their poor stability, high sensitivity toward water, and inherent moisture due to the use of organic ammonium cations has restricted their commercialization. Interestingly, rhenium cluster cations are much heavier than organic ammonium cations, and thus using a rhenium cluster as a cation could improve the long-term stability of hybrid materials. Furthermore, hybrid crystals containing rhenium cluster cations and inorganic anions can exhibit synergetic broad-band optical properties. Electronic interactions between the anionic inorganic lead halide framework and emissive rhenium cluster cations can give rise to unusual photophysical properties. As shown in this report, this unique 1D structure results in strong broad-band bathochromically shifted emission, peaking at 634 nm with a large Stokes shift.

## Experimental section

### Materials

Rhenium, sulfur, bromine, cesium bromide, 1*H*-pyrazole-1-carboxamidine hydrochloride (99%), lead(ii) iodide (99.999% trace metals basis, perovskite grade), methylammonium iodide (MAI) (99%, anhydrous), and *N*,*N*-dimethylformamide (DMF) (anhydrous, 99.8%) were purchased from Sigma Aldrich and used without additional purification. Polymeric precursor Re_6_S_8_Br_2_ was prepared *via* high-temperature solid-state synthesis from elemental Re and S powders and Br_2_ liquid.

### Preparation of Cs_4_[Re_6_S_8_Br_6_]·2H_2_O

The starting cluster compound Cs_4_[Re_6_S_8_Br_6_]·2H_2_O was synthesized from Re_6_S_8_Br_2_ as described previously.^34^ In detail, stoichiometric quantities of metallic rhenium, sulfur, and cesium bromide were ground, thoroughly mixed, and loaded into a quartz ampoule. The ampoule was evacuated, and bromine liquid was added. The mixture was frozen with liquid nitrogen, and the ampoule was evacuated and sealed. The sealed ampoule was heated to 850 °C at the rate of 0.5 °C min^−1^, held at this temperature for 72 h, and then cooled to room temperature at 6 °C min^−1^. The product was washed with water and then with acetone, and then dried in an oven at 80 °C for 24 h.

### Preparation of [Re_6_S_8_(PzH)_6_]Cl_2_ (I)

Base on the synthesis of [Re_6_S_8_(PzH)_6_]Cl_2_ where PzH is pro-ligands – pyrazole described previously,^35^ 1*H*-pyrazole-1-carboxamidine hydrochloride was used instead of pyrazole as a source of pyrazole ligands and chloride anions. In the details, Cs_4_[Re_6_S_8_Br_6_]·2H_2_O of 0.2 g (0.083 mmol) and excess 1*H*-pyrazole-1-carboxamidine hydrochloride of 0.2 g (1.36 mmol) were ground in a mortar, and the resulting mixture was allowed to react by heating it in a sealed glass ampoule at 200 °C for two days. The ampoule was then slowly cooled to room temperature and opened. The reaction mixture was washed with diethyl ether to remove excess ligand and then washed with water to remove unreacted starting cluster compounds and the reaction by-product cesium bromide. The remaining solid was dried in an oven at 80 °C for 24 h to give the corresponding yellowish rhenium cluster complex in a yield of 80%. Crystals suitable for X-ray structure determination were obtained directly from the mixture after the reaction. Energy Dispersive X-ray Spectroscopy (EDS) showed a consistent Re : S : Cl ratio of 6.0 : 6.4 : 2.1 (Fig. S1†). Anal. calc. for C_18_H_24_Cl_2_N_12_Re_6_S_8_ (1853.10): C, 11.7; H, 1.3; N, 9.1; S, 13.8. Found: C, 12.27; H, 1.12; N, 10.08; S, 11.13%. (The discrepancy may cause from solvate PzH molecules because the powder obtained usually contains solvate PzH molecules.)

### Preparation of {[Re_6_S_8_(PzH)_6_][Pb_3_I_8_(DMF)_2_]}·6(DMF) (II)

Specific stoichiometric quantities of PbI_2_ (0.75 g. 1.62 mmol), MAI (0.17 g, 1.08 mmol), and [Re_6_S_8_(PzH)_6_]Cl_2_ (1 g, 0.54 mmol) were dissolved in 40 ml of DMF and allowed to react at 80 °C for 24 h with vigorous stirring under flowing argon gas. The resulting solution was filtered before growing single crystals. Crystals suitable for X-ray structure determination were obtained by means of diffusion of diethyl ether into a solution of the mixture in DMF. Orange rod-shaped crystals were obtained after three weeks. The yield is about 28.9%. EDS shows a consistent Re : S : Pb : I ratio of 6.0 : 8.3 : 3.2 : 7.5 (Fig. S2†). LC-MS (negative mode) contains a few sets of intense peaks in the area *m*/*z* 818–819 (Fig. S3†). The most intense peak can be attributed to the [Pb_3_I_8_]^2−^ anion (*m*/*z* = 818.42). Anal. calc. for C_42_H_80_I_8_N_20_O_8_Pb_3_Re_6_S_8_ (4003.79): C, 12.6; H, 2.0; N, 7.0; S, 6.4%. Found: C, 11.6; H, 1.7; N, 7.6; S, 5.3%. (The discrepancy may cause from solvate PzH molecules.)

### Instrumentation

Absorption spectra were obtained by means of UV-vis spectrometry. Powder X-ray diffraction (PXRD) measurements were collected using a Bruker D8-Focus Bragg-Brentano X-ray powder diffractometer with a Cu Kα radiation source (*λ* = 1.5418 Å). PXRD data were acquired over the range 5–60°. The emission spectra of solid {[Re_6_S_8_(PzH)_6_][Pb_3_I_8_(DMF)_2_]}·6(DMF) was measured on a Perkin Elmer LS 55 fluorescence spectrometer equipped with a xenon lamp at 298 K. Emission spectra were recorded over the range 400–900 nm with an excitation wavelength of 340 nm. EDS measurements were recorded on a SEM-EDS (JEOL, Tokyo, Japan). LC-MS (liquid chromatography-mass spectrometry) was taken on Ultimate 3000 RSLC (Thermo scientific) and Q-exactive orbitrap plus MS (Thermo scientific) in negative condition.

### Time-resolved photoluminescence (TR-PL) experiments

Time-resolved PL decay data for all crystal dispersion samples were recorded using a home-built TR-PL decay measurement system. It utilizes an EKSPLA tunable nanosecond pulse laser (NT-342), a Hamamatsu microchannel plate PMT (R3809U-50) detector, Jovin-Yvon H10VIS monochromator, and Tektronix TDS 3052B 500 MHz digital oscilloscope. The typical pulse width of the excitation beam is about 3 ns, and the power is managed to be less than ∼100 μJ per pulse. The instrument response function (IRF) appeared to be almost a delta function in the current experiments, therefore no deconvolution was necessary for date fitting processes. TR-PL decay data were acquired from a home-built software developed using Labview (National Instruments) and all the solution samples were prepared in a 1 cm quartz cuvette by dissolving crystal samples in dimethylformamide (DMF, anhydrous 99.8%, Sigma Aldrich) at ambient conditions.

### X-ray crystallography

X-ray diffraction data for I and II were collected on a Bruker APX-II diffractometer equipped with a monochromator in an Mo Kα (*λ* = 0.71073 Å) incident beam. Each crystal was mounted on a glass fiber, and the data for I and II were collected at 223 and 170 K, respectively. The CCD data were integrated and scaled using the Bruker-SAINT software package, and the structure was solved and refined using SHEXL-2014. All hydrogen atoms were placed in the calculated positions. Table 1 lists the crystallographic data for I and II. Tables S1 and S2† list selected bond distances and angles for compounds I and II, respectively. Structural information was deposited at the Cambridge Crystallographic Data Centre; the CCDC reference numbers are 2034482 for I and 2034483 for II.†

**Table 1:** Crystal data and structure refinements for I and II

	I		II	
Empirical formula	Re_6_S_8_(PzH)_6_Cl_2_		{[Re_6_S_8_(PzH)_6_][Pb_3_I_8_(DMF)_2_]}·6DMF	
Formula weight	1853.10		4003.71	
Temperature	223(2) K		170(2) K	
Wavelength	0.71073 Å		0.71073 Å	
Crystal system	Monoclinic		Triclinic	
Space group	*P*2_1_/*c*		*P*1̄	
Unit cell dimensions	*a* = 11.0944(6) Å	*α* = 90°	*a* = 10.5488(5) Å	*α* = 90.416(3)°
	*b* = 16.4078(10) Å	*β* = 99.941(3)°	*b* = 11.2290(5) Å	*β* = 93.892(3)°
	*c* = 20.5808(12) Å	*γ* = 90°	*c* = 19.1582(10) Å	*γ* = 97.737(2)°
Volume	3690.2(4) Å^3^		2243.18(19) Å^3^	
*Z*	4		1	
Density (calculated)	3.335 Mg m^−3^		2.964 Mg m^−3^	
Absorption coefficient	20.234 mm^−1^		16.654 mm^−1^	
*F*(000)	3312		1784	
Crystal size	0.090 × 0.040 × 0.040 mm^3^		0.300 × 0.060 × 0.040 mm^3^	
Theta range for data collection	1.597 to 24.998°		1.830 to 28.331°	
Index ranges	−13 ≤ *h* ≤ 13, −19 ≤ *k* ≤ 19, −24 ≤ *l* ≤ 24		−13 ≤ *h* ≤ 14, −14 ≤ *k* ≤ 14, −25 ≤ *l* ≤ 25	
Reflections collected	94 398		72 160	
Independent reflections	6454 [*R*(int) = 0.0509]		11 031 [*R*(int) = 0.0761]	
Data/restraints/parameters	6454/8/413		11 031/0/373	
Goodness of fit on *F*^2^	1.225		1.035	
Final *R* indices [*I* > 2*σ*(*I*)]	*R*_1_ = 0.0369, w*R*_2_ = 0.0705		*R*_1_ = 0.0472, w*R*_2_ = 0.1247	
*R* indices (all data)	*R*_1_ = 0.0566, w*R*_2_ = 0.0838		*R*_1_ = 0.0611, w*R*_2_ = 0.1348	
Largest diff. peak and hole	1.742 and −1.530 e Å^−3^		3.885 and −4.795 e Å^−3^	

## Results and discussion

### Crystal structures

In the preparation of [Re_6_S_8_(PzH)_6_]Cl_2_ (I), all six apical Br ligands of the starting cluster Cs_4_[Re_6_S_8_Br_6_]·2H_2_O were substituted by pyrazole coordinated through the nitrogen of the pyrazole ring, producing the apically homoleptic cationic complex [{Re_6_S_8_}(PzH)_6_]^2+^. During reaction process at high temperature, 1*H*-pyrazole-1-carboxamidine can be decomposed to release pyrazole ring. However, according to stoichiometry the reaction mixture contains 0.498 mmol of Br^−^-anions and 1.36 mmol of Cl^−^-anions. Thus the excess of Cl^−^-anions leads to the formation of [Re_6_S_8_(PzH)_6_]Cl_2_ with chlorine as anions. [Re_6_S_8_(PzH)_6_]Cl_2_ (I) has been previously prepared,^35^ but its crystal structure is determined for the first time. The crystal structure of I is isostructural to the selenium analogue [Re_6_Se_8_(PzH)_6_]Cl_2_.^35^

**Fig. 1:**
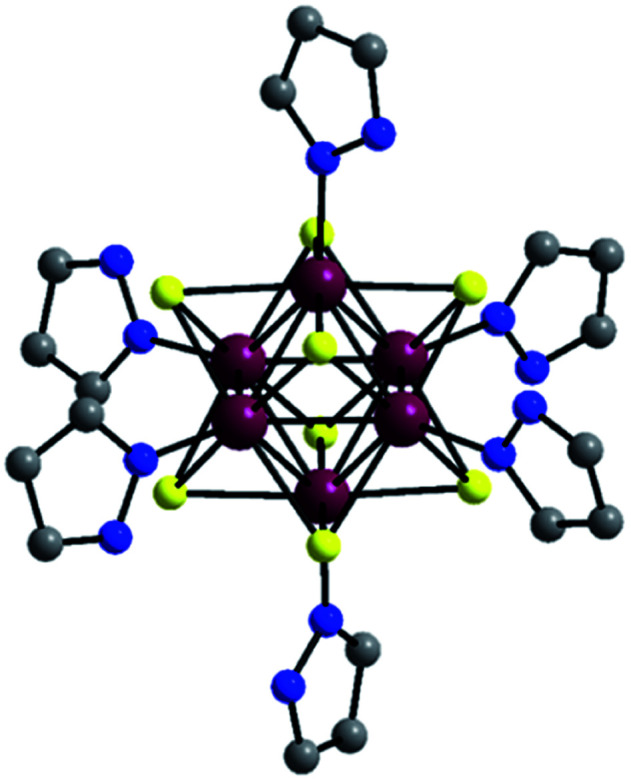
Structure of [{Re_6_S_8_}(PzH)_6_]^2+^. All hydrogen atoms and disordered atoms are omitted for clarity. Atom colors: Re, purple; S, yellow; N, blue; C, grey.

The compound [Re_6_S_8_(PzH)_6_]Cl_2_ crystallizes in a monoclinic cell with space group *P*2_1_/*c* (Table 1). The structure of the cluster cationic unit [{Re_6_S_8_}(PzH)_6_]^2+^ is shown in Fig. 1. The cationic complex [{Re_6_S_8_}(PzH)_6_]^2+^ contains {Re_6_S_8_}^2+^ which consists of a Re_6_ octahedron residing inside a S_8_ cube; that is to say, each face of the Re_6_ octahedron is coordinated by a μ_3_-S-bridged ligand. This is typical for 24e Re_6_ cluster complexes like [Re_6_(μ_3_-Q)_8_L_6_] having 12 two-electron metal–metal bonds. Each Re is additionally coordinated by the nitrogen atom of a pyrazole. Average interatomic distances within the cluster unit are 2.5926(5) Å, 2.412(2) Å, and 2.166(8) Å for the Re–Re, Re–S, and Re–N bonds, respectively (Table S1†).

The 1D lead iodide hybrid compound {[Re_6_S_8_(PzH)_6_][Pb_3_I_8_(DMF)_2_]}·6(DMF) (II) was prepared by dissolving specific stoichiometric quantities of PbI_2_, MAI, and the rhenium cluster [Re_6_S_8_(PzH)_6_]Cl_2_ (I) in DMF at 80 °C for 24 h with vigorous stirring under flowing argon gas. A typical wet chemical synthetic method for preparing low-dimensional perovskites involves dissolution of the organic ammonium salt and lead halide salt in a suitable solvent by heating. In the present work, the emissive and air-stable rhenium cluster complex was used for the first time as the cation, instead of using an organic halide salt, and both the rhenium cluster and the lead iodide were dissolved in DMF. Diethyl ether was added to the clear DMF solution to provide single crystals of II for X-ray crystallographic analysis. All crystals were dried under vacuum and used for further characterizations.

**Fig. 2:**
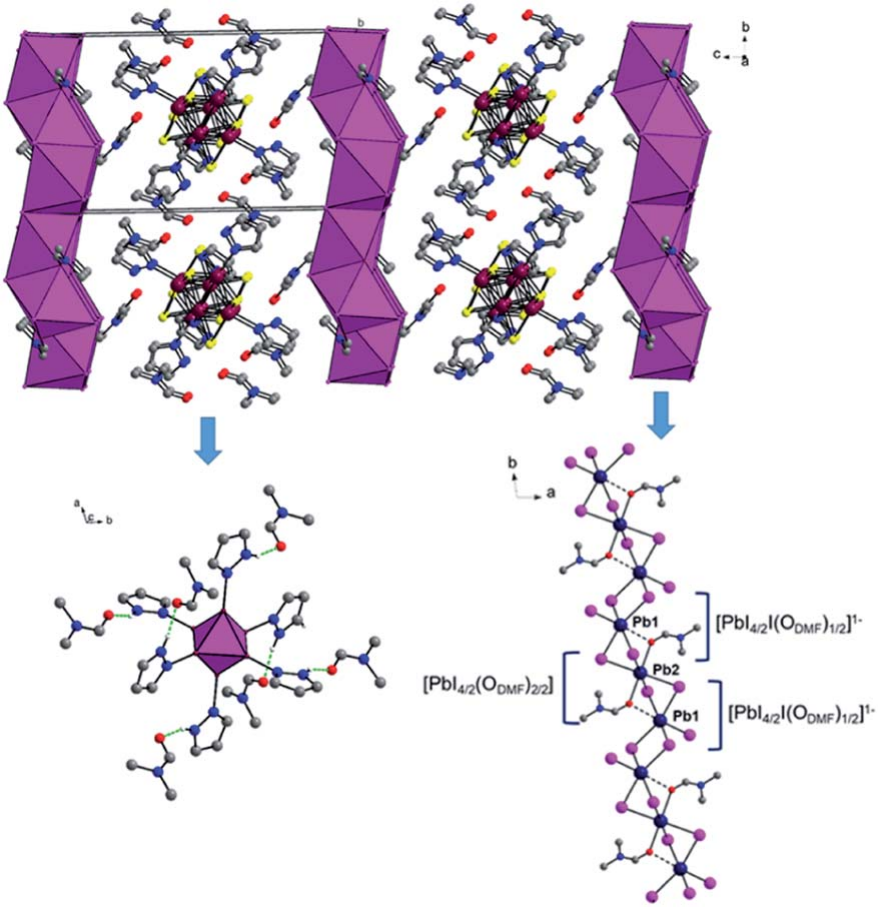
1D structure of [Re_6_S_8_(PzH)_6_][Pb_3_I_8_(DMF)_2_]·6(DMF) along the *a* axis. Lead iodides are shown as polyhedra. 1D lead iodide chain containing a lead iodide set, {[PbI_4/2_I(DMF)_1/2_][PbI_4/2_(DMF)_2/2_][PbI_4/2_I(DMF)_1/2_]}^2−^. Each pyrazole N–H of Re_6_S_8_^2+^ is connected to DMF oxygen atom through H-bonds (green dotted lines). The S atoms were omitted for clarity, and Re_6_S_8_^2+^ is shown as a polyhedron. All hydrogen atoms except pyrazole N–H are omitted for clarity. Atom colors: Re, purple; S, yellow; N, blue; C, grey; O, red; Pb, dark blue; I, pink.

The structure of II is depicted in Fig. 2, and crystallographic data are given in Table 1. The 1D lead iodide hybrid formulated as {[Re_6_S_8_(PzH)_6_][PbI_3_I_8_(DMF)_2_]}·6(DMF) crystalized in orange rods with the triclinic space group of *P*1̄. The crystal structure is shown in Fig. 2. Two DMF oxygen atoms bridge three lead iodides to form a set of lead iodides, {[PbI_4/2_I(O_DMF_)_1/2_][PbI_4/2_(O_DMF_)_2/2_][PbI_4/2_I(O_DMF_)_1/2_]}^2−^, and these sets of lead iodide share edges to form a 1D lead iodide chain, [Pb_3_I_8_(DMF)_2_]^2−^. The middle lead atom in a set of lead iodide is octahedrally bound to four iodides and two apical DMF oxygen atoms, and the other two lead atoms are also octahedrally bound to five iodides and one elongated Pb–O_DMF_ bond. The Pb–O_DMF_ distance is 2.577 Å (solid lines) and the elongated Pb–O_DMF_ is 3.013 Å (dotted lines in Fig. 2). This 1D lead iodide chain has never been reported before and is different from the typical edge-sharing octahedral PbI_6_ units. The 1D lead iodide chains are stacked along the *a* axis, and [Re_6_S_8_(PzH)_6_]^2+^ cations and free DMF molecules reside between the 1D chains. Each pyrazole N–H of [Re_6_S_8_]^2+^ is connected to DMF oxygen atom through H-bonding (N_PzH_–H⋯O_DMF_ 1.82–1.88 Å) as shown in green dotted lines (Fig. 2).

**Fig. 3:**
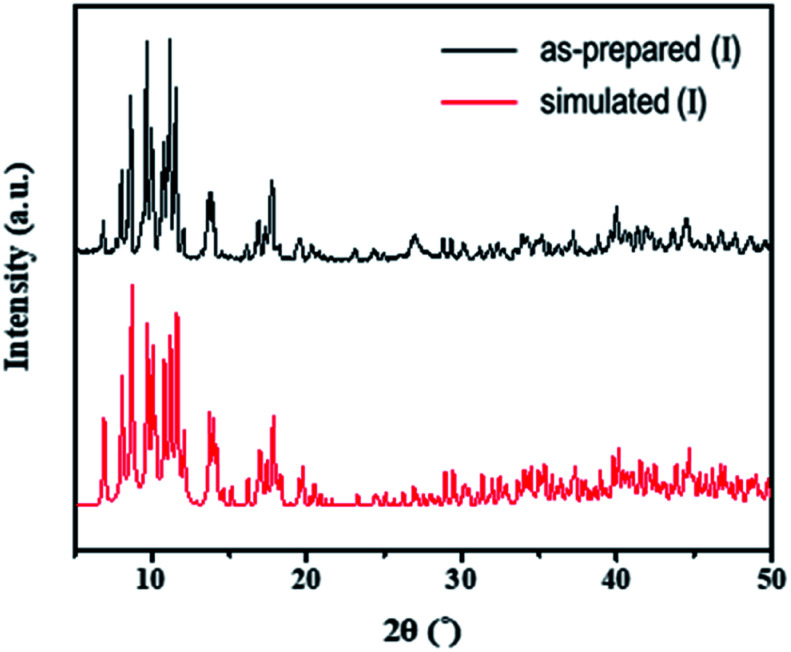
Observed PXRD patterns of the as-prepared (I) and simulated PXRD patterns based on the single-crystal structure.

**Fig. 4:**
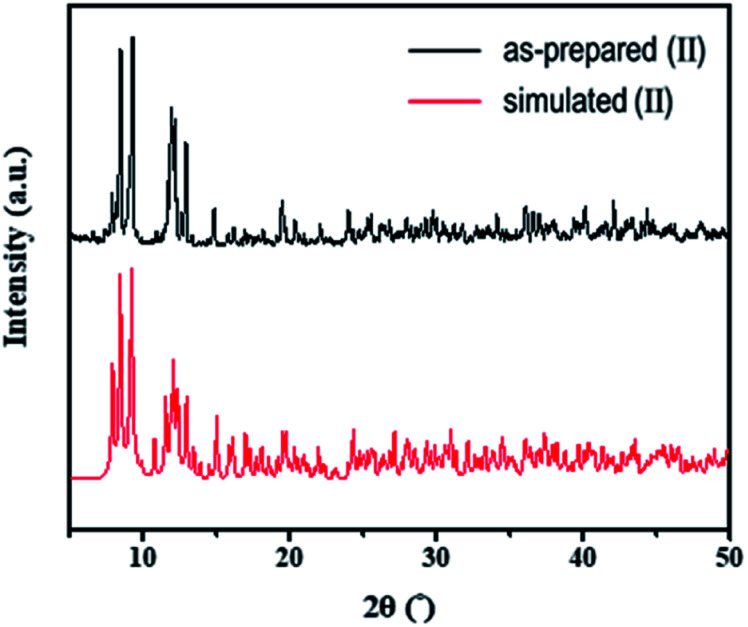
Observed PXRD patterns of as-prepared (II) and simulated PXRD patterns based on the single-crystal structure.

The structures of both the prepared rhenium cluster (I) as a cation precursor and the 1D lead iodide hybrid (II) were again confirmed by means of powder X-ray diffraction analysis. The PXRD patterns of the as-prepared I and II were in good agreement with the simulated PXRD patterns (Fig. 3 for I and Fig. 4 for II). The disagreement in the intensity between some PXRD peaks can be attributed to differences in preferred orientation due to the 1D structure.

**Fig. 5:**
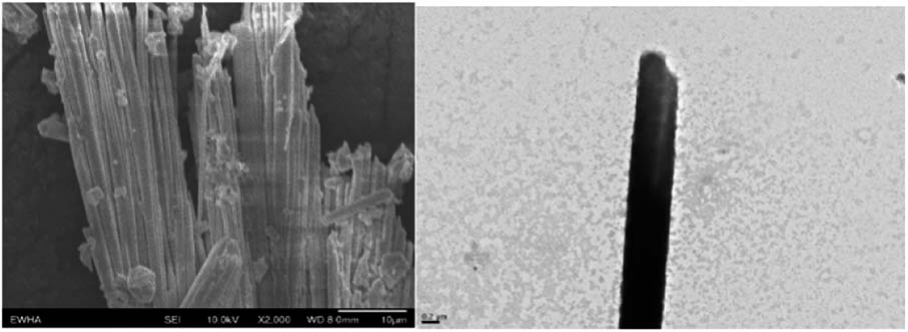
SEM images of the 1D lead iodide hybrid.

Scanning electron microscopy (SEM) images of the 1D lead iodide hybrid are shown in Fig. 5. A large number of microrods were obtained in II. This crystal shape is totally different from the microplate shape of the starting rhenium cluster compound (I) (Fig. S4†). The length of the microrods ranged from several to tens of micrometers.

**Fig. 6:**
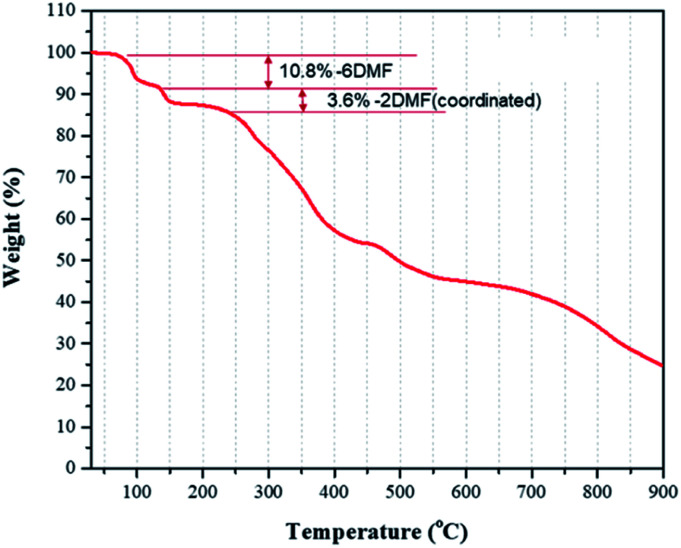
TGA data for weight loss of 1D lead iodide hybrid (II).

TGA analysis of crystals of II showed three stages of weight losses (Fig. 6). In the range from 90 to 150 °C, a weight loss of about 10% was attributed to a loss of solvate DMF molecules (calc. 10.8%), and a loss about 3% was attributed to loss of coordinated DMF ligands (calc. 3.59%).

### Optical properties

Both rhenium cluster complex (I) and 1D lead iodide hybrid (II) dissolved in DMF were excited with laser pulses at the wavelength of 340 nm and optical measurements were carried out at 298 K. Emission spectra were recorded over the wavelength range 400–900 nm. Absorption, excitation and emission of I are shown in Fig. S5.† The emission spectra of compounds I and II in both solution and solid state are similar as shown in Fig. S6.†

**Fig. 7:**
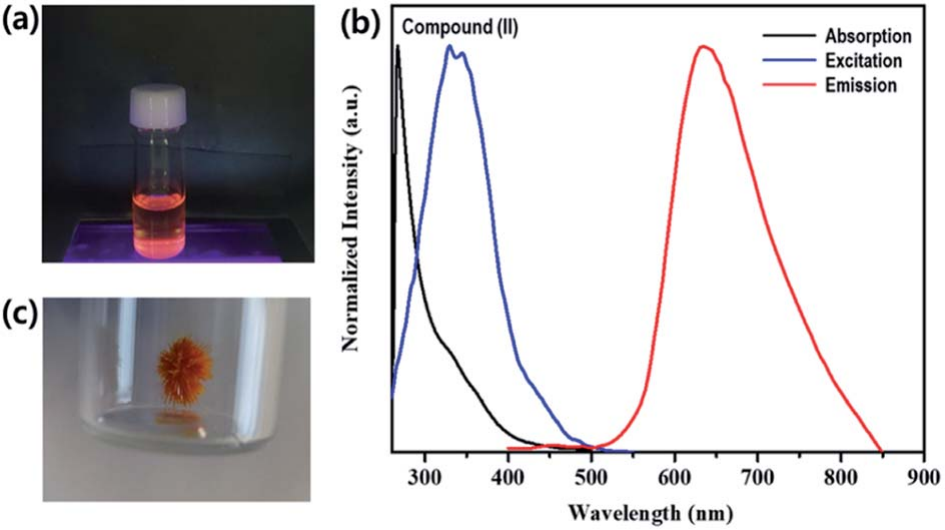
(a) Photograph of 1D lead iodide hybrid (II) in DMF under UV light (365 nm). (b) Absorption, excitation and emission of (II) in DMF solution. (c) Photograph of a crystal of (II) under ambient light.

**Fig. 8:**
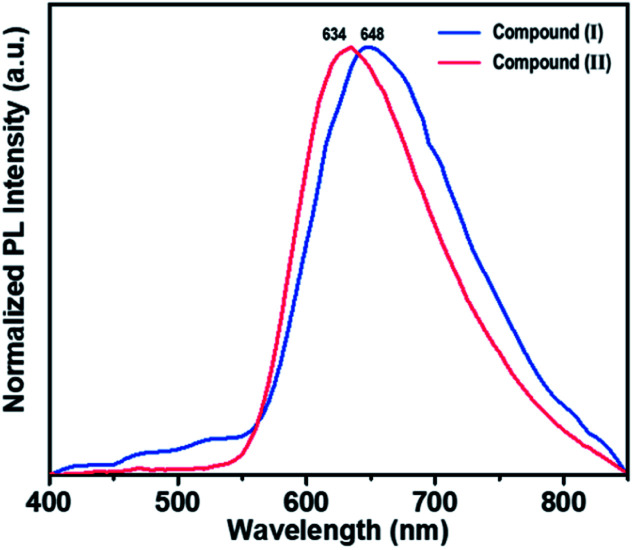
Emission spectra of I and II in solid state.

Brown needle-shaped crystals of II are shown in Fig. 7c. Under UV irradiation (*λ* = 365 nm), the II compound in DMF exhibited a bright orange emission (Fig. 7a). To further characterize the optical properties of II, its UV-vis absorption and photoluminescence (PL) spectra were acquired (Fig. 7b). The UV-vis absorption spectrum displayed sharp absorption ranging from 200 to 450 nm and a weak broad absorption band at about 330 nm, possibly caused by an intramolecular π electron transition in conjugated organic ligand attached hexarhenium cations.^36^ Upon excitation at 340 nm, the 1D lead iodide hybrid (II) had a broad band that peaked at 634 nm and spanned from 550 nm to 850 nm, covering the entire visible range (Fig. 7). The luminescence spectra for both I and II were measured in solid state (Fig. 8). Characteristic emission of the {Re_6_S_8_} core with maximum at 648 nm (blue line in Fig. 8) was observed in compound (I) which was assigned to the rhenium cluster, and the maximum of compound (II) at 634 nm (red line in Fig. 8) has been bathochromically shifted. This result indicates that 1D lead iodide contained hybrid compound (II) displays broad-band light emission similar to that of rhenium cluster core and a larger bathochromically shifted emission due to lead iodide anions overlapped with that of rhenium cluster in 550 range shifting maximum peak of Re cluster to shorter wavelength from 648 nm to 634 nm as shown Fig. 8.

Absorption and emission spectra of the 1D lead halide hybrid showed a large Stokes shift of 310 nm. In 2017, Yuan and coworkers reported one-dimensional organic lead bromide perovskites (C_4_N_2_H_14_PbBr_4_) that showed broad emission with a maximum at 475 nm.^21^ In 2019, Biswas and coworkers synthesized a one-dimensional perovskite [(H_2_O) (C_6_H_8_N_3_)_2_Pb_2_Br_10_] that also gave an efficient bluish white emission at around 560 nm.^37^ More recently, a series of one-dimensional face-sharing perovskites were reported by Feng and coworkers; these exhibited broad-band bluish white light emissions with maximum peaks around 500 nm.^36^ In the present work, the emission spectrum of a 1D lead halide chain wrapped by rhenium cluster cations showed a strongly enhanced bathochromically shifted emission compared with reported 2D and 1D lead halide perovskites including ammonium cations, which usually exhibit characteristic peaks in the violet to green region.^22,23,38^

**Fig. 9:**
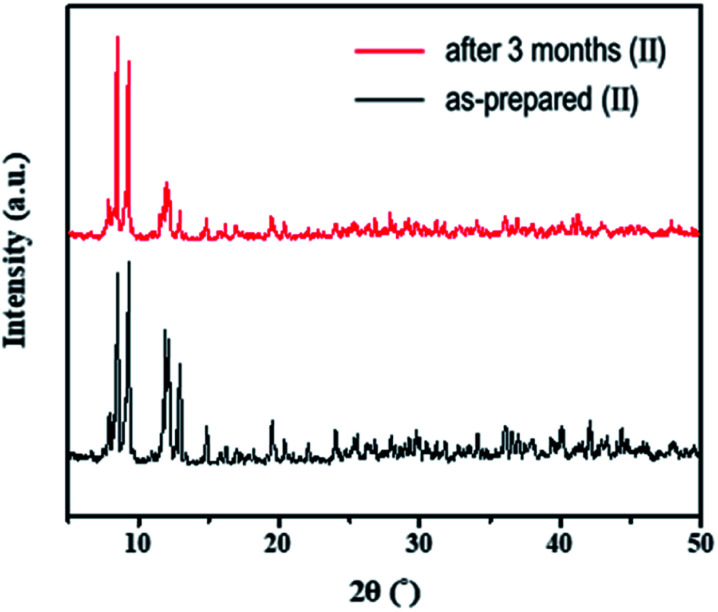
PXRD patterns of {[Re_6_S_8_(PzH)_6_][Pb_3_I_8_(DMF)_2_]}·6(DMF) (II) after 3 months.

To test the moisture tolerance of compound (II), a solution of II in DMF was exposed to air at room temperature for 3 months. The emissions of compound (II) are originated from [Re_6_S_8_(PzH)_6_]^2+^ and [Pb_3_I_8_(DMF)_2_]^2−^ in solution. We found that the shape of emissions of solution was not changed after three months indicating no decomposition of two luminophores [Re_6_S_8_(PzH)_6_]^2+^ and [Pb_3_I_8_(DMF)_2_]^2−^. The stability of the solid phase of {[Re_6_S_8_(PzH)_6_][Pb_3_I_8_(DMF)_2_]}·6(DMF) was also confirmed by powder X-ray diffraction as shown in Fig. 9. This evidence shows that the 1D lead iodide hybrid is stable in ambient atmosphere.

**Fig. 10:**
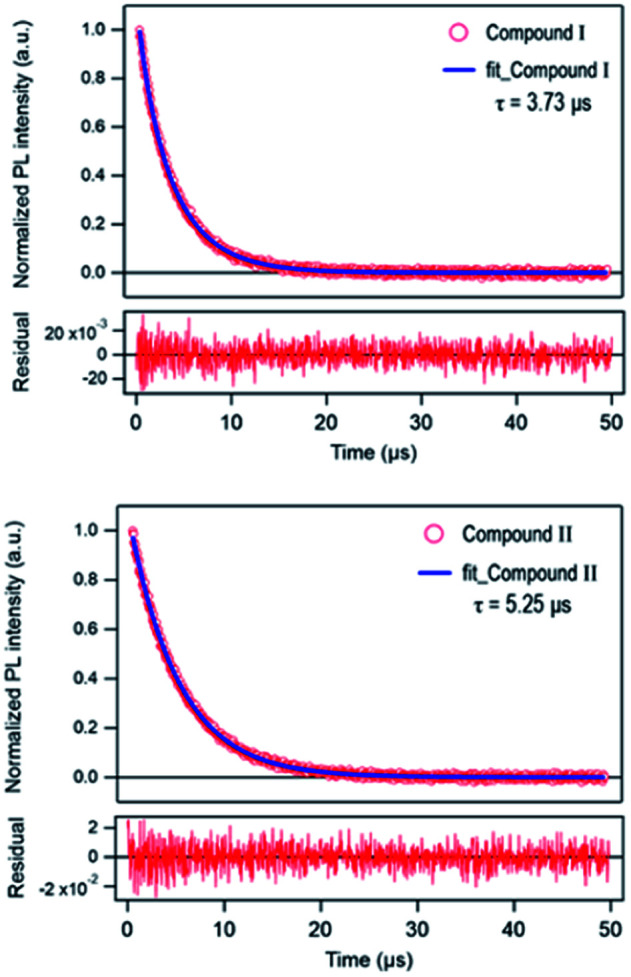
Time-resolved PL decay curve of [Re_6_S_8_(PzH)_6_]Cl_2_ (I) and {[Re_6_S_8_(PzH)_6_][Pb_3_I_8_(DMF)_2_]}·6(DMF) (II) at 300 K.

**Fig. 11:**
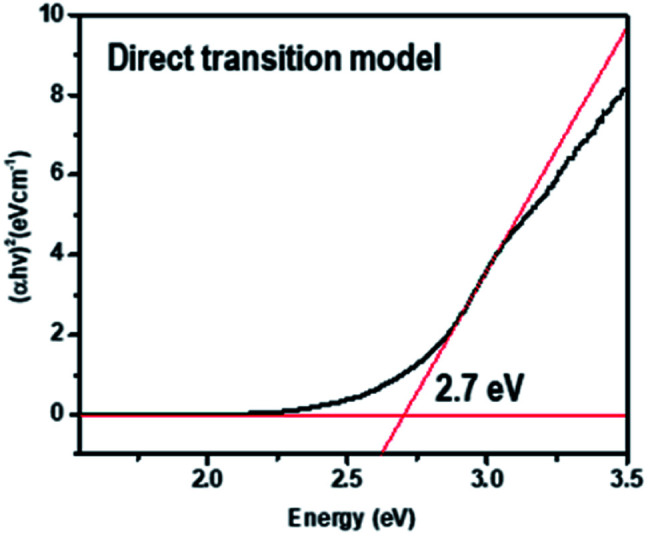
Tauc plot for {[Re_6_S_8_(PzH)_6_][Pb_3_I_8_(DMF)_2_]}·6(DMF) (II).

The time-resolved 650 nm PL decay curve of compound (II) collected at 300 K was fitted by a double exponential function, giving the average PL lifetime of 5.25 μs, and this was an increased value from 3.77 of compound (I) (Fig. 10). Finally, the band gap was calculated by using Tauc's function on both indirect and direct transition models. Tauc plots gave the best fitting for the direct band gap model; thus, the band gap of compound (II) obtained by the direct transition model is about 2.7 eV (Fig. 11). This band gap is characteristic for low-dimensional perovskite materials^39^ and the excitonic absorption was stable at room temperature. The band gap was similar to that reported for other 1D perovskites such as CsPbI_3_ (3.05 eV)^40^ and [tetrabutylammonium][PbI_3_] (2.76 eV).^41^

## Conclusions

Using for the first time a rhenium cluster cation as a structure-directing spacer and a luminophore for an organometallic lead halide, we were able to design and synthesize the novel lead iodide hybrid {[Re_6_S_8_(Pz)_6_][Pb_3_I_8_(DMF)_2_]}·6(DMF) with structural architectures based on 1D chains. This 1D lead iodide contained hybrid compound (II) displays broad-band light emission similar to that of rhenium cluster core and a larger bathochromically shifted emission due to lead iodide anions overlapped with that of rhenium cluster in 550 range shifting maximum peak of Re cluster to shorter wavelength from 648 nm to 634 nm. Specifically, the emission peaked near 634 nm, an unprecedentedly long wavelength for the lead halide hybrid. This work verifies that there is a strong synergetic interaction between anions and cation clusters favorable for exciton self-trapping to produce highly efficient broad-band luminescence. This compound's highly efficient and stable broad-band light emissions are promising for use in light emitters for optoelectronic devices.

This report opens a new path for the synthesis of optically useful materials combining optically active inorganic cations and anions. For the first time, the rhenium cluster cation was used as a cation to stabilize lead iodide chains, and this material will pave the way to a new class of compounds with great potential for application in photonic devices.

## Author contributions

The original idea was conceived by G. T. L. and S.-J. K.; experiments and data analysis were performed by G. T. L., J. C., Y. K., S. K. and S.-H. Y.; the crystal structure of the 1D lead iodide hybrid was analyzed by Y. K.; and the manuscript was drafted by G. T. L., J. C., Y. K. and S.-J. K. All authors analyzed the data and reviewed and contributed to the manuscript.

## Conflicts of interest

There are no conflicts to declare.

## Supplementary Material

RA-011-D1RA04170C-s001

RA-011-D1RA04170C-s002
