# Supplementary material for: One-dimensional lead iodide hybrid stabilized by inorganic hexarhenium cluster cations as a new broad-band emitter
Source: RSC Adv. 2021 Jul 13;11(40):24580–7. doi: 10.1039/d1ra04170c (PMC9036903; doi:10.1039/d1ra04170c)
Supplement: RA-011-D1RA04170C-s001 [file RA-011-D1RA04170C-s001.pdf]

Electronic Supplementary Material (ESI) for RSC Advances.
This journal is © The Royal Society of Chemistry 2021

Supporting information

One-dimensional lead iodide hybrid stabilized by inorganic hexarhenium cluster cations as new broad band emitter

Giang Thi Ly†, Jun Choi†, Youngmee Kim, Yuna Kim, Sujin Kim, Sohyeon Yang, and Sung-Jin Kim*

Department of Chemistry and Nanoscience, Ewha Womans University, Seoul, 03760, Republic of Korea

† These authors contributed equally.

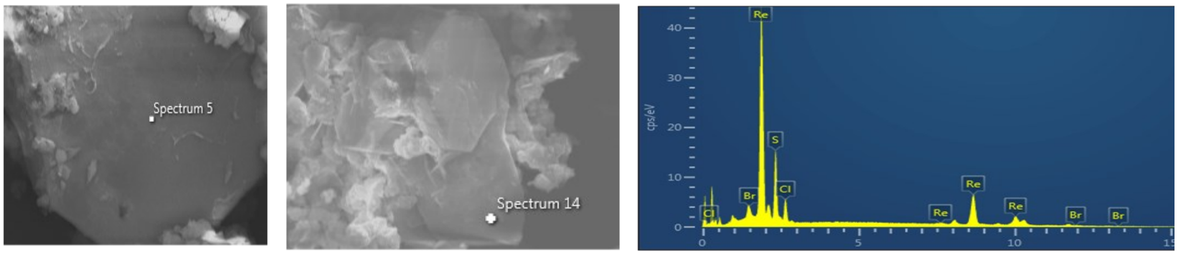

Element	Atomic %	Ratio
**S**	44.02	6.4
**Cl**	14.17	2.1
**Re**	41.04	6.0

Fig. S1. EDS determination of $[Re_6S_8(PzH)_6]Cl_2$ (**I**)

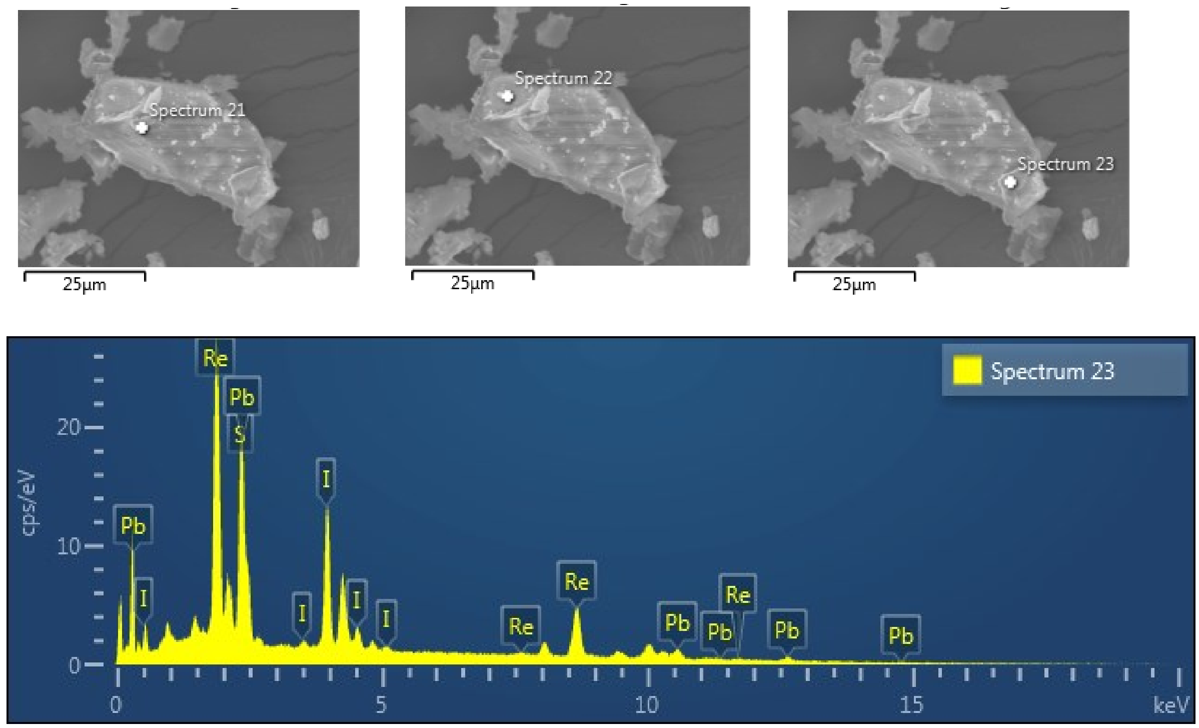

Element	Atomic %	Ratio
**S**	33.13	8.3
**I**	29.90	7.5
**Re**	24.08	6.0
**Pb**	12.89	3.2

Fig. S2. EDS analysis confirms the presence of the constituent elements in the $\{[Re_6S_8(PzH)_6][Pb_3I_8(DMF)_2]\}\cdot 6(DMF)$ (**II**).

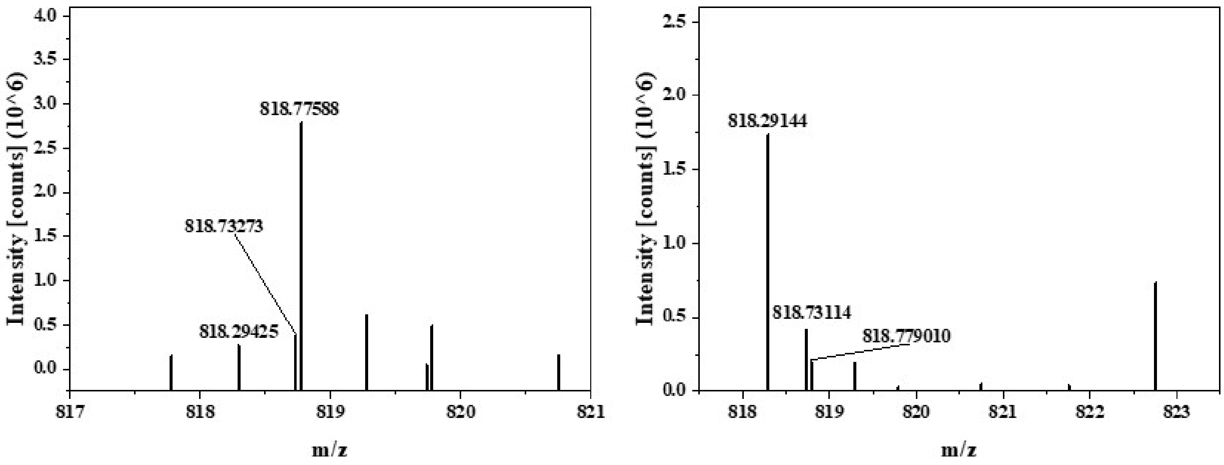

Fig. S3. LC-MS (negative mode) of **II** contains a few sets of intense peaks in the area *m/z* 818-819. The most intense peak can be attributed to the $[Pb_3I_8]^{2-}$ anion (*m/z* = 818.42).

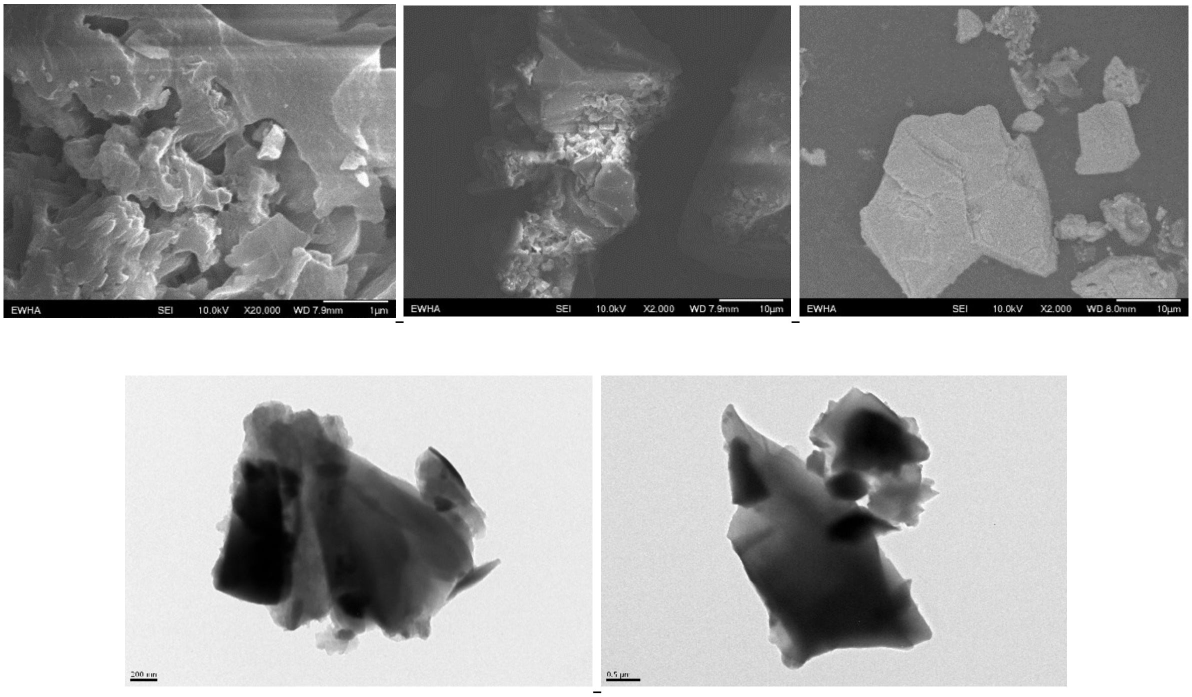

Fig. S4. SEM images of $[Re_6S_8(PzH)_6]Cl_2$ (**I**).

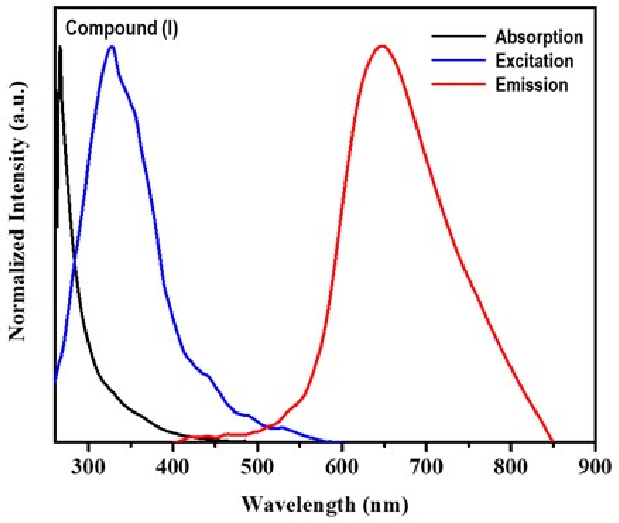

Fig. S5. Absorption, excitation and emission of **I** in DMF solution.

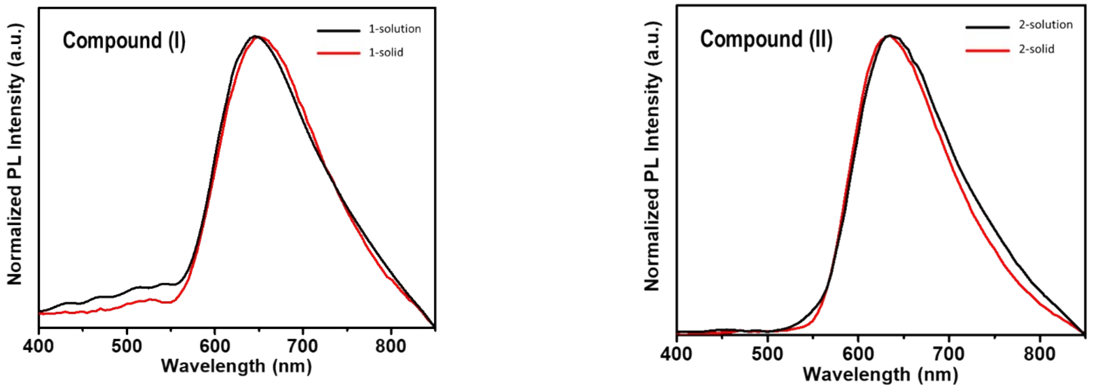

Fig. S6. Emission spectra of **I** and **II** in solid state and in DMF solution.

Table S1. Selected bond lengths of **I**.

Re(1)-N(1)	2.144(12)
Re(1)-S(1)	2.422(3)
Re(1)-S(8)	2.422(3)
Re(1)-S(2)	2.429(3)
Re(1)-S(7)	2.433(3)
Re(1)-Re(4)	2.5815(7)
Re(1)-Re(6)	2.5818(7)
Re(1)-Re(5)	2.5829(7)
Re(1)-Re(2)	2.5919(8)
Re(2)-N(3)	2.192(12)
Re(2)-S(4)	2.423(3)
Re(2)-S(2)	2.428(3)
Re(2)-S(1)	2.428(3)
Re(2)-S(3)	2.436(3)
Re(2)-Re(3)	2.5813(7)
Re(2)-Re(6)	2.5884(7)
Re(2)-Re(5)	2.5896(7)
Re(3)-N(5)	2.158(11)
Re(3)-S(6)	2.422(3)
Re(3)-S(3)	2.427(3)
Re(3)-S(5)	2.430(3)

Re(3)-S(4)	2.431(3)
Re(3)-Re(5)	2.5882(7)
Re(3)-Re(4)	2.5908(7)
Re(3)-Re(6)	2.5948(7)
Re(4)-N(7)	2.157(12)
Re(4)-S(8)	2.424(3)
Re(4)-S(6)	2.424(3)
Re(4)-S(5)	2.427(3)
Re(4)-S(7)	2.435(3)
Re(4)-Re(6)	2.5821(7)
Re(4)-Re(5)	2.5885(7)
Re(5)-N(9)	2.146(11)
Re(5)-S(1)	2.422(3)
Re(5)-S(6)	2.423(3)
Re(5)-S(7)	2.428(3)
Re(5)-S(4)	2.431(3)
Re(6)-N(11)	2.169(11)
Re(6)-S(5)	2.411(3)
Re(6)-S(8)	2.416(3)
Re(6)-S(3)	2.424(3)
Re(6)-S(2)	2.426(3)

Table S2. Selected bond lengths of **II**.

Re(1)-N(11)	2.159(8)
Re(1)-S(1)	2.408(2)
Re(1)-S(2)	2.413(2)
Re(1)-S(4)	2.414(2)
Re(1)-S(3)	2.414(2)
Re(1)-Re(2)#1	2.5856(5)
Re(1)-Re(3)#1	2.5875(5)
Re(1)-Re(2)	2.5899(5)
Re(1)-Re(3)	2.5916(5)
Re(2)-N(21)	2.150(8)
Re(2)-S(3)#1	2.406(2)
Re(2)-S(4)#1	2.410(2)
Re(2)-S(1)	2.410(2)
Re(2)-S(2)	2.421(2)
Re(2)-Re(1)#1	2.5856(5)
Re(2)-Re(3)	2.5871(5)
Re(2)-Re(3)#1	2.5875(5)
Re(3)-N(31)	2.144(9)
Re(3)-S(1)#1	2.403(2)
Re(3)-S(3)	2.404(2)
Re(3)-S(4)#1	2.412(2)

Re(3)-S(2)	2.412(2)
Re(3)-Re(2)#1	2.5876(5)
Re(3)-Re(1)#1	2.5876(5)
S(1)-Re(3)#1	2.403(2)
S(3)-Re(2)#1	2.406(2)
S(4)-Re(2)#1	2.409(2)
S(4)-Re(3)#1	2.412(2)
Pb(1)-I(3)	2.9281(11)
Pb(1)-I(4)	3.1232(12)
Pb(1)-I(4)#2	3.1345(12)
Pb(1)-I(1)	3.2389(10)
Pb(1)-I(2)	3.3101(9)
Pb(2)-O(41)	2.577(9)
Pb(2)-O(41)#3	2.577(9)
Pb(2)-I(2)	3.2085(8)
Pb(2)-I(2)#3	3.2086(8)
Pb(2)-I(1)#3	3.2540(9)
Pb(2)-I(1)	3.2540(9)
I(4)-Pb(1)#2	3.1345(12)

Symmetry transformations used to generate equivalent atoms:

#1 -x,-y+1,-z+1 #2 -x+2,-y,-z #3 -x+2,-y+1,-z
